# Profiling the Concentration of Reduced and Oxidized Glutathione in Rat Brain Using HPLC/DAD Chromatographic System

**DOI:** 10.3390/molecules26216590

**Published:** 2021-10-30

**Authors:** George Jîtcă, Erzsébet Fogarasi, Bianca-Eugenia Ősz, Camil Eugen Vari, Ibolya Fülöp, Mircea Dumitru Croitoru, Carmen Maria Rusz, Maria Titica Dogaru

**Affiliations:** 1 Department of Pharmacology and Clinical Pharmacy, Faculty of Pharmacy, George Emil Palade University of Medicine, Pharmacy, Science and Technology of Târgu Mureș, 540139 Târgu Mureș, Romania; george.jitca@umfst.ro (G.J.); bianca.osz@umfst.ro (B.-E.Ő.); camil.vari@umfst.ro (C.E.V.); maria_dogaru2004@yahoo.com (M.T.D.); 2 Doctoral School of Medicine and Pharmacy, I.O.S.U.D., George Emil Palade University of Medicine, Pharmacy, Science and Technology of Târgu Mureș, 540139 Târgu Mureș, Romania; carmenrusz20@gmail.com; 3 Department of Toxicology and Biopharmacy, Faculty of Pharmacy, George Emil Palade University of Medicine, Pharmacy, Science and Technology of Târgu Mureș, 540139 Târgu Mureș, Romania; ibolya.fulop@umfst.ro (I.F.); mircea.croitoru@umfst.ro (M.D.C.)

**Keywords:** oxidative stress, glutathione, brain, liquid chromatography, diode array detector

## Abstract

This study aimed to develop a HPLC/DAD method in order to determine and quantify the reduced glutathione (GSH) and oxidized glutathione (GSSG) levels in rat brain. Due to the presence of the thiol group (-SH), GSH can interact with the Ellman′s reagent (DTNB), with which it forms a reaction product through which the level of GSH can be quantified, using the DAD detection system. Chromatographic separation was achieved after a derivatization process by using a mobile phase acetonitrile (A) and phosphate buffer (20 mM, pH = 2.5) (B). The compounds of interest were detected at 330 nm using a chromatographic C8 column. The method of determination met the validation criteria, specified by the regulatory bodies. The applicability of the method was demonstrated in a chronic toxicology study of central nervous system (CNS), following different treatment regimens with haloperidol.

## 1. Introduction

Glutathione (GSH) is a tripeptide (l-γ-glutamyl-l-cysteinyl-glycine) that exhibits many biological roles, including the protection against reactive oxygen and nitrogen species (ROS and RNS). Thus, the GSH antioxidant pathway is one of the most important and well-represented components of the endogenous antioxidant system [1]. At the same time, the cysteine residue, reactive thiol (-SH) group, present in its chemical structure, is responsible for the antioxidant effect by neutralizing the ROS and RNS [2,3].

GSH is involved in many processes, such as defense and preservation of the organism, prevention or delay of age-related diseases onset, given the proportional increase of free radicals with aging [4]. It is found mainly in its reduced form (GSH) because the enzyme responsible for converting the oxidized form (GSSG) is induced by oxidative stress. Therefore, GSH/ GSSG ratio is considered an indicator of oxidative status [5,6]. It has been observed that the inherited enzyme defects related to GSH are very rare; in contrast, the inbalances in GSH homeostasis associated with the increase of the oxidative stress levels in central nervous system (CNS) are common in neurodegenerative diseases [7].

Low levels of GSH/ GSSG ratio are incriminated in the onset of diseases such as cancers [8,9], diabetes [10], CNS disorders: Parkinson’s, Alzheimer’s disease [11,12,13], or disorders that appear following psychotropic drug abuse [14]. GSH also accomplishes a variety of functions at cellular level, which could explain the correlation between the depletion of this antioxidant molecule and the pathogenesis of neurodegenerative diseases.

In order to determine the concentrations of GSH, different analytical methods were applied: spectrophotometry [15], liquid chromatography tandem mass spectrometry (LC-MS) [16], or gas chromatography-mass spectrometry (GC-MS) [17]. Despite their advantages in terms of preparation time or analysis speed, there are important disadvantages that cannot be disregarded: lack of specificity (spectrophotometry) or high costs.

Along with malondialdehyde, which is another important marker of oxidative stress [18], the quantification of GSH/GSSG ratio can be used in estimating the oxidative status in multiple genetic or drug-induced diseases. To avoid the bias generated by the interaction between Ellman’s reagent (5,5′-dithio-bis(2-nitrobenzoic) acid, DTNB) and another molecule (cysteine), and with the attempt to obtain as close GSH and GSSG values as the real ones, this study aims to validate a simple method of determination of GSH and GSSG levels in brain, with applicability in preclinical studies.

**Figure 1:**
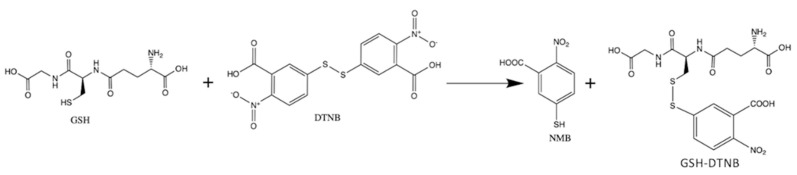
Reaction of Glutathione (GSH) with Ellman’s reagent/ DTNB (5,5′-dithio-bis-(2-nitrobenzoic acid)); 2-Nitro-5-mercapto-benzoic acid (NMB).

The chromatographic analysis of the analytes resulting from GSH with DTNB reaction (Figure 1) was performed with a high-performance liquid chromatographic (HPLC) system coupled with a diode array detector (HPLC/DAD). The main disadvantage of the photometric technique, frequently employed for the GSH measurement in biological samples, is the lack of specificity, because other thiol containing molecules, such as cysteine for example, could lead to false readings [19]. Furthermore, in photometric techniques it is difficult to obtain a high-quality blank, since both the reagent used and the sample can have a certain interfering color. Another disadvantage of the photometry used for GSH measurements is the low detectability, along with a narrow limit of concentrations that can precisely and accurately be measured. Due to these reasons, a chromatographic technique, such as the HPLC, could solve these problems by improving the detectability and the concentration range that can be used without further sample preparation process (dilution for example). Furthermore, with the possibility of separating interfering substances and the use of DAD "peak purity" test, the specificity could also be increased. From Figure 1 it can be seen that thiol interference could not be also avoided by HPLC, if the 2-Nitro-5-mercapto-benzoic acid (NMB) is measured as in the photometric technique. However, by following the GSH-DTNB peak, which lacks visible light absorption but has UV light absorption properties, a very good specificity could be attained, since even thiol interference could be avoided by chromatographic separation.

Indeed, more time and higher costs could be needed for the chromatographic technique, but the significant improvement in the detectability, concentration, and the possibility to detect a GSH specific compound rather than a -SH specific compound justifies the importance of this research.

## 2. Results

In this analythical method, validation parameters are represented by: linearity, selectivity, accuracy, precision, lower limit of quantification (LLOQ), stability, and robustness. All of these parameters were verified in accordance with the guidelines presented by the regulatory bodies (FDA 2018) [20].

### 2.1. Chromatographic Conditions

The chromatographic separation of GSH, after GSH derivatization, was evaluated by using a mobile phase consisted of acetonitrile (A) and phosphate buffer (20 mM, pH = 2.5) (B). The elution gradient is presented in Table 1. The injection volume was 50 µL and a retention time of 6.22 ± 0.06 was obtained. The flow rate was set at 1 mL/min and the analytes were detected at 330 nm by a DAD (range: 200–700 nm) detector using a chromatographic column Zorbax Eclipse XDB-C8, 5 µm, 4.6 × 150 mm. The GSH-DTNB reaction occurs at room temperature in 10 min. The reduction of GSSG occurs at 80 °C after 60 min of incubation in the presence of DTNB.

### 2.2. Linearity and LLOQ

**Figure 2:**
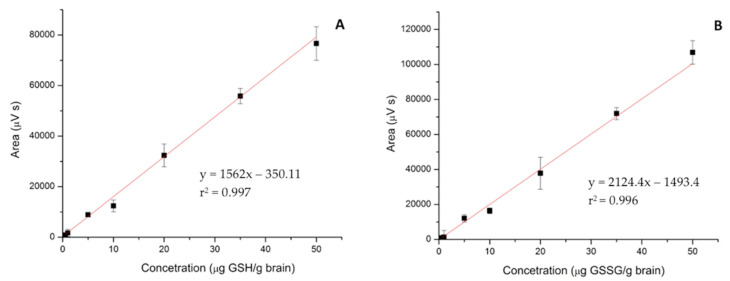
Analytical curves for reduced (GSH) (**A**) and oxidized glutathione (GSSG) (**B**) in the range 0.50–50 µg/g brain.

The linearity of the method was verified using an analytical curve on seven concentration levels, evaluated in triplicates. The derivatization reaction was performed for each level of concentration used for the analytical curve. The analytical curve for the GSH is described by the linear equation: y = 1562x − 350.11 with a determination coefficient r^2^ = 0.997 (Figure 2A), while analytical curve for GSSG is described by the linear equation: y = 2124.4x − 1493.4, and r^2^ = 0.996 (Figure 2B). y represents the analyte peak area and x is the concentration, as shown in Table 2.

### 2.3. Selectivity

**Figure 3:**
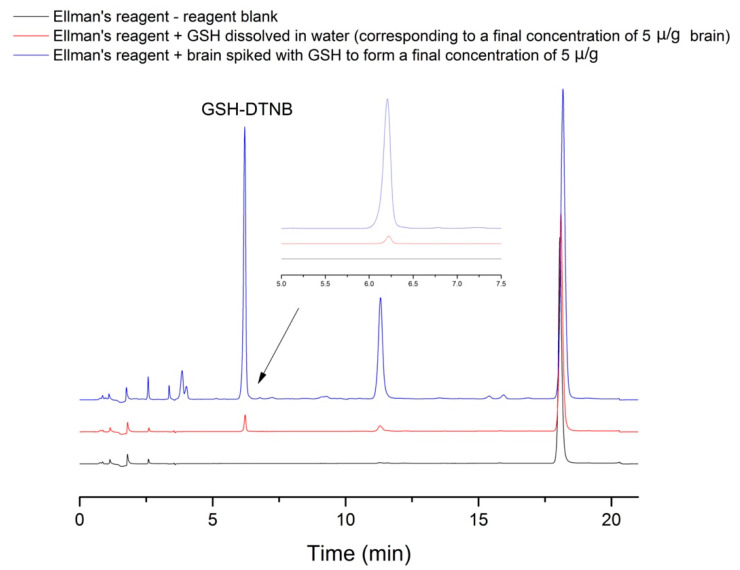
Representative chromatograms of a spiked rat brain sample.

In order to verify the selectivity, three samples were injected, prepared in triplicates according to the procedure described in Section 4.3. Sample preparation with the following modification: DTNB without GSH, DTNB with GSH, and brain sample with GSH and DTNB. At the retention time of the GSH-DTNB complex there are no overlapping peaks; the peak of interest has a corresponding purity, as can be seen in Figure 3.

### 2.4. Accuracy

Quality control (QC) samples at LLOQ concentration were spiked in order to determine accuracy and precision. For each QC level, five replicates were analyzed in one run for the intra-day procedure. 

Accuracy was evaluated based on GSH percentage and GSSG percentage recovered from the matrix. Results of accuracy for the intra- and inter-day precision for GSH and GSSG at LLOQ and QC levels are presented in Table 3.

### 2.5. Precision

Concerning the evaluation of inter-day precision, it has been evaluated in two different days, using five replicates for each QC level and at LLOQ concentration, respectively. The intra- and inter-day precision results were expressed as RSD%. The results for GSH and GSSG at LLOQ and three QC levels are presented in Table 3.

Both for intra- and inter-day analysis, the precision (RSD%) of QC samples was ≤15% and the accuracy ranged ±15%. These results demonstrated the fact that the method is reproducible for the determination of GSH and GSSG in rodent’s brain, considering that accuracy and precision were found to be within acceptable limits.

### 2.6. Stability

The stability of the QC samples was stored at room temperature for 12 h and 24 h, respectively, both in five replicates was assessed; brain samples were stored at −80 °C prior to the analysis. For GSH samples, the analytical recovery varied between 87.18–114.78% after 12 h and between 94.86–118.29% after 24 h at room temperature (25 °C). For GSSG samples, the analytical recovery varied between 87.88–111.45% after 12 h. The results are illustrated in Table 4.

### 2.7. Robustness

The robustness of the method was evaluated with the performance of variations in two important chromatographic parameters: mobile phase pH and flow rate, which were modified throughout the analysis. All of the tests were performed at three levels of concentration of 0.5, 15, and 40 µg/g for GSH in five replicates. Results are listed in Table 5.

## 3. Discussion

Measurement of GSH in biological samples is of high importance in the study of oxidative stress and exogenous substances or pathological conditions associated with oxidative state [21,22].

It is well known that GSH has a low stability in the biological samples, because after the blood or the tissue is devoided by the normal oxygen supply, reactive species are formed, and those species could quickly oxidize the available reduced glutathione (GSH). This process makes the measurement of GSH in biological samples a challenge. Frequently, GSH is photometrically measured following a derivatization with the Ellman′s reagent that forms a non-specific colored compound with the thiol groups [23]. Due to this fact, one can be certain on measuring thiol groups, not only GSH. In our paper, we developed an HPLC-UV method that was able to detect not the generally formed colored compound by the thiol groups with the Ellman’s reagent, but the non-colored but UV absorbing compound formed after this reaction.

Our method has significant advantages over the most commonly used photometric method in the following parameters:

- specificity: using photometry we can be sure about the thiol molecule content of the sample, not GSH in a specific way. Furthermore, photometry does not easily allow a double blank: native color of the sample + native color of the reagents. This problem can be achieved by column separation;
- detectability: lower limits of GSH can be detected by the use of the HPLC method;

The increased temperature leads to the reduction of oxidized glutathione (GSSG), and reaction of the newly formed GSH with the Ellman’s reagent is possible by reacting the brain sample with the Ellman’s reagent at room temperature and high temperature (about 80 °C) to measure the GSSG content of the sample. This is an important step in evaluating the oxidative stress level, not because the absolute value of the GSH and GSSG is important but because of the ratio of the two components [24]. After the method development, on a number of 40 rats we measured the native GSH and GSSG content and the GSH/GSSG ratio.

The performance of the analytical method (validation) was measured according to international requirements [20]; therefore, one can consider valid any brain GSH/GSSG evaluation made with our method.

Indeed, sample preparation in the HPLC technique is not less time consuming than the photometric method, but is certainly less “sample consuming”. This makes room for analysis of very low quantity samples. Furthermore, the more time needed for the chromatographic analysis compared with the very fast and easy reading on a photometer is well compensated by the increased specificity and detectability that we have shown during our method validation.

GSSG does not react with Elman’s reagent directly. However, at high temperature (80 °C), Elman’s reagent reduces GSSG to GSH, and then the same reaction as in the case of GSH–Ellman’s reagent occurs. The reduction ratio of GSSG to GSH has high importance when GSSG is desired to be measured in biological samples. Reduction ratio of GSSG to GSH was measured after heating the samples for 60 min at 80 °C.

The newly analytical method, developed by our team, was succesfully applied in detection of GSH and GSGG from rat brain samples in a pharmacological experiment that can change the level of the oxidative stress in rodent’s brain. Average concentrations of GSH and GSSG in untreated (blank) animals was 24.82 ± 1.68 µg/g brain and 6.54 ± 3.09 µg/g brain, respectively. It can be seen that the newly developed method is able to easily and precisely quantify the concentration of the compound of interest in the desired biological matrix.

## 4. Materials and Methods

### 4.1. Chemicals and Reagents

All chemicals and reagents used in this study were of analytical purity, being purchased from different providers: acetonitrile was purchased from VWR International, SAS, Fontenay-sous-Bois, France, anhydrous disodium phosphate (Na_2_HPO_4_), and 85% phosphoric acid solution (H_3_PO_4_) were purchased from Merck KGaA (Darmstadt, Germany). The Ellman’s reagent, trichloroacetic acid (TCA), ethylenediaminetetraacetate (EDTA-Na_2_) powder, sodium chloride (NaCl) powder, phosphate buffer solution (PBS), GSH and GSSG powders were all purchased from Sigma-Aldrich (Darmstadt, Germany). Ultra pure water was obtained using the Milli-Q purification system (Merck Millipore Corporation, Burlington, MA, USA).

### 4.2. Preparation of Solutions

For GSH stock solution (5 mg/mL) preparation, 5 mg of reduced glutathione were dissolved in 1 mL of ultra pure water. GSSG stock solution (5 mg/mL) was obtained by dissolving 5 mg of oxidized glutathione in 1 mL of ultra pure water.

Working solutions for GSH and GSSG at 9 levels of concentration (25, 50, 250, 500, 750, 1000, 1500, 2000, 2500 μg/mL) were obtained by succesive dillution of the stock solutions with ultra pure water. Seven (GSH, GSSG) triplicate samples for linearity (0.5-50 μg/g) and three QC samples were prepared (LLOQ, 15 μg/g, 40 μg/g) in five replicates.

The derivatization reaction with the Ellman’s reagent was applied individually to each sample, after the preparation of the calibration curve solutions.

### 4.3. Sample Preparation

With the aim of obtaining as accurate results as possible, for optimization of sample preparation and avoiding unnecessary prolongation of sample preparation which could influence the final results, we applied an automated homogenization method using the IKA Ultra-Turrax Tube [18].

Twenty Wistar male rats, weighing 400–500 g, were placed in individual plastic cages. The animals were maintained on a 12:12 h light dark cycle and fed ad libitum. Afterwards, the animals were decapitated under anesthesia with ketamine and xylazine in a dose mixture of ketamine (100 mg/kg) and xylazine (10 mg/kg) in order to collect the brain samples. After brain samples extraction, these were immediately immersed in liquid nitrogen and stored at −80 °C until analysis.

In order to analyze GSH and GSSG, brains were homogenized using IKA Ultra-Turrax Tube Drive and then divided in equal quantities. 1g of each GSH and GSSG brain sample was then spiked with 10 μL of working solution, and then 3 mL of PBS were added. Samples were vortexed for 1 min, and immediately after they were centrifuged (10,000× *g* for 10 min). Following centrifugation, 500 µL supernatant was collected and 500 µL Ellman’s reagent was added to the supernatant. The GSSG samples were then heated for 60 min at 80 °C in the TS-100S, Thermo-Shaker (BioSan, Riga, Latvia). The GSH samples were not subjected to the heat-treatment, but instead they were left at room temperature for 10 min.

To both GSH and GSSG series of samples, 300 μL TCA 20% was added and then the samples were centrifuged at 13,000× *g* for 10 min. After centrifugation, the supernatant was collected and transferred into HPLC vials.

### 4.4. Determination of the Degree of Reduction of Oxidized Glutathione

Following the reaction between Ellman’s reagent and GSH, a GSH-DTNB reaction product is obtained. Thus, the quantification of GSSG levels requires the calculation of the difference between the total glutathione (TG) and GSH. The determination of TG can be done by reducing GSSG to GSH.

This method presents and advantage of chemical nature from the sample preparation point of view, because the reduction of GSSG will occur in the presence of Ellman’s reagent at 80 °C, without the necessity of additional reducing compunds and preparation steps. The validation of this reduction process required the preparation of the samples in three different conditions:

1. Condition 1: Biological samples spiked with GSSG heated at 80 °C for 60 min;
2. Condition 2: Biological samples spiked with GSH heated at 80 °C for 60 min;
3. Condition 3 (control condition): Biological samples spiked with GSH stored at room temperature for 10 min;

The calculated differences between the two conditions (Condition 1 and 2) reflect the degree of GSSG reduction. The reduction degree of GSSG to GSH is presented in Table 6.

### 4.5. Instrumentation

The validation of the present method was performed using a HPLC Merck system: quaternary pump Merck Hitachi L-7100, auto sampler Merck Hitachi L-7200, column thermostat Merck Hitachi L-7360, DAD Merck Hitachi L-7455, interface Merck Hitachi L-7000, solvent degaser Merck Hitachi L-7612, software D-7000 HSM-Manager (Hitachi Corporation, Westford, MA, USA)

### 4.6. Study Application

The method was succesfully applied in a chronic CNS toxicity study, following different haloperidol treatment regimens, in order to demonstrate the applicabilty. The brains were rapidly removed, frozen in liquid nitrogen, and stored at −80 °C. The preparation of the samples was performed according to the steps presented in Section 4.2 Preparation of Solutions, and for the analysis, the discussed method was applied. 

### 4.7. Ethical Considerations

All of the experimental procedures were in accordance with the European Directive 2010/63/UE and the study was granted the approval of the Ethics Committee of Scientific Research of the George Emil Palade University of Medicine, Pharmacy, Science and Technology of Târgu Mureș (approval no. 533/2019) and National Sanitary Veterinary and Food Safety Authority (approval no. 42/2020).

## 5. Conclusions

In this article, an analytical method for quantifying the level of GSH and GSSG in the neural matrix (brain) was validated. In this regard, the analysis was performed using a C8 type chromatographic column and an elution gradient. Regarding the validation parameters examined, they were found to be in accordance with the regulatory guidelines and regulations (FDA 2018).

The developed method in this study is applicable in research studies that focus on quantification of GSH and GSSG levels in rodent brains, as markers of oxidative stress, with different etiologies.

## Figures and Tables

**Table 1:** Brief presentation of the mobile phase elution gradient program.

Time (min)	Phosphate Buffer, 20 mM, pH = 2.5 (%)	Acetonitrile (%)	Flow (mL/min)
0	98	2	1
19	50	50	1
19.1	98	2	1
21	98	2	1

**Table 2:** Validation results of the analytical factors of HPLC method.

Analytical Factor	GSH	GSSG
LLOQ (µg/g brain)	0.50	
rLLOQ (%)	97.11	98.86
LLOQrec (µg/g brain)	0.50	0.50
rLLOQrec (%)	85.47	108.78
Slope	1562	2124.4
Y-intercept	−350.11	−1493.4
Determination coefficient (r^2^)	0.997	0.996
Analytical range (µg/g brain)	0.50–50	
Retention time	6.22 ± 0.06	

rLLOQ, relative lower limit of quantification; LLOQrec, recovery corrected LLOQ; rLLOQrec (%), relative recovery-corrected LLOQ;

**Table 3:** Accuracy and precision of reduced glutathione (GSH) and oxidized glutathione (GSSG) in lower limits of quantification and quality control samples.

Conc. (µg/g Brain)		Intra-Day			Inter-Day		
		Mean	RSD %	Accuracy %	Mean	RSD %	Accuracy %
GSH	0.5	0.57	1.47	113.62	0.57	1.62	113.85
	15	14.27	6.73	95.15	13.28	6.56	88.54
	40	45.12	7.69	112.79	45.33	7.04	113.32
GSSG	0.5	0.45	0.15	90.88	0.49	9.74	97.45
	15	16.73	3.33	111.55	15.72	14.52	104.81
	40	44.03	5.79	110.08	42.48	13.38	106.20

**Table 4:** Stability assessment for samples stored at room temperature for 12 and 24 h, respectively.

Parameters		Stability for Samples Stored at Room Temperature					
		Conc. (µg/g Brain)					
		0.5		15		40	
		12 h	24 h	12 h	24 h	12 h	24 h
GSH	Mean	0.63	0.54	16.39	16.89	39.34	43.97
	Rec *, %	110.04	94.86	114.78	118.29	87.18	97.44
	RSD%	4.92	10.17	5.41	0.34	12.23	2.26
GSSG	Mean	0.51	-	14.71	-	87.88	-
	Rec *, %	111.45	-	87.88	-	92.93	-
	RSD%	0.52	-	4.27	-	8.33	-

* Recovery, average of three concentrations.

**Table 5:** Robustness of the method by variation of two chromatographic parameters (mobile phase ratio and mobile phase pH value).

	Conc. (µg/g Brain)	Retention Time (min) ± RSD, %	Peak Purity (%) ± RSD, %
Mobile phase pH value			
2.3	0.5	6.95 ± 0.06	99.25 ± 1.12
	15	6.95 ± 0.05	98.03 ± 2.12
	40	6.95 ± 0.04	98.16 ± 0.99
2.5	0.5	6.24 ± 0.03	96.42 ± 1.22
	15	6.21 ± 0.08	99.81 ± 0.78
	40	6.21 ± 0.06	99.68 ± 0.73
2.7	0.5	6.96 ± 0.04	98.16 ± 1.36
	15	6.95 ± 0.03	98.98 ± 1.43
	40	6.95 ± 0.09	98.64 ± 2.43
Flow (mL/min)			
0.9	0.5	7.80 ± 0.02	98.37 ± 1.27
	15	7.78 ± 0.10	99.17 ± 2.54
	40	7.77 ± 0.09	98.98 ± 2.77
1.0	0.5	6.24 ± 0.05	99.36 ± 2.44
	15	6.21 ± 0.05	98.13 ± 1.87
	40	6.21 ± 0.08	99.07 ± 2.89
1.1	0.5	6.72 ± 0.06	99.15 ± 1.72
	15	6.68 ± 0.03	99.67 ± 1.99
	40	6.64 ± 0.03	98.17 ± 2.48

**Table 6:** Determination of the degree of reduction of oxidized glutathione.

Conc. (μg/g Brain)	Percentage of Reduction (%)	SD (+/− %)
0.5	108.79	7.80
1	90.19	6.33
5	111.10	9.72
10	113.91	10.49
15	114.60	4.64
20	112.35	9.38
35	113.35	11.29
40	103.40	7.74
50	91.66	10.31

## Data Availability

The datasets that support the findings of this study are available from the corresponding authors upon reasonable request.

## Sample Availability

Samples of the compounds are not available from the authors.
